# Application of Photodynamic Therapy in Cardiology

**DOI:** 10.3390/ijms25063206

**Published:** 2024-03-11

**Authors:** Piotr Wańczura, David Aebisher, Maksymilian Kłosowicz, Angelika Myśliwiec, Klaudia Dynarowicz, Dorota Bartusik-Aebisher

**Affiliations:** 1 Department of Cardiology, Medical College, The Rzeszów University, 35-310 Rzeszów, Poland; 2 Department of Photomedicine and Physical Chemistry, Medical College, The Rzeszów University, 35-310 Rzeszów, Poland; 3 English Division Science Club, Medical College, The Rzeszów University, 35-310 Rzeszów, Poland; klosowiczmaksymilian@gmail.com; 4 Center for Innovative Research in Medical and Natural Sciences, Medical College, The Rzeszów University, 35-310 Rzeszów, Poland; amysliwiec@ur.edu.pl (A.M.); kdynarowicz@ur.edu.pl (K.D.); 5 Department of Biochemistry and General Chemistry, Medical College, The Rzeszów University, 35-310 Rzeszów, Poland; dbartusikaebisher@ur.edu.pl

**Keywords:** ablation, cardiology, atherosclerosis, reactive oxygen species, photodynamic therapy

## Abstract

The origins of photodynamic therapy (PDT) date back to 1904. Since then, the amount of research proving PDT and, consequently, its applicability to various disease states has steadily increased. Currently, PDT is mainly used in oncology to destroy cancer cells. It is being worked on for possible use in other medical fields as well, including cardiology. It can be used in the prevention of restenosis, often occurring after vascular surgical interventions, for destroying atherosclerotic plaques and as a new ablative method of ectopic centers in the treatment of atrial fibrillation. The purpose of this review is to summarize the knowledge to date regarding the therapeutic potential of using PDT for various pathological conditions in cardiology. The review also focuses on the current limitations associated with the use of PDT and identifies areas where more research is needed to develop better drug regimens. Materials and methods: The study analyzed 189 medical articles. The articles came from PubMed, Frontiers, Google Scholar, Science Direct and Web of Science databases. Through the excitation of light, a photosensitizer (PS) introduced into the body, the destruction of pathological cells occurs. PTD is widely used in oncology of the central nervous system (CNS). This process is made possible by the production of free oxygen radicals (ROS) and singlet oxygen, which generate oxidative stress that destroys sensitive cancer cells. In recent years, photosensitizers have also been discovered to have a strong affinity for macrophages that fill atherosclerotic plaques, making these compounds suitable for treating atherosclerosis. By inducing apoptosis of smooth muscle cells, inactivating basic fibroblast growth factor (FGF-β) and inhibiting endothelial cell hyperplasia, PDT can be used to prevent restenosis after surgical proceduresPDT appears to be a minimally invasive and highly effective therapeutic method, especially when combined with other therapeutic methods. Unfortunately, the small number of animal model studies and human clinical trials greatly limit the applicability of PDT on a wider scale. Current limitations, such as the depth of penetration, delivery of photosensitizer particles to the direct site of the lesion or the appropriate choice of photosensitizer in relation to the nature of the pathology, unfortunately make it impossible to replace current therapeutic approaches.

## 1. Introduction

The first reports of photodynamic therapy (PDT) date back to 1904, when Raab and Tappeiner observed that certain dyes, introduced into the body, could cause cell death under the influence of the applied light beam. In 1948, 44 years later, Figge proved that porphyrins—now common photosensitizers (PSs)—tend to be stored in tumor cells in mice [1]. The topic of cancer treatment with PDT has been developed by subsequent researchers from generation to generation [2]. Currently, PDT appears to be a promising method for treating mainly cancerous conditions [3,4]. It is being studied for its possible therapeutic effects in the treatment of acne and age-related macular degeneration, as well as bacterial, fungal and viral infections [5,6,7,8]. In recent years, more and more reports have appeared in the field of cardiology, showing that PDT can be used to stabilize myocardial plaques, in the prevention of restenosis after surgical corrections and in ablations of ectopic rhythm-generating centers in the treatment of atrial fibrillation (AF) [9,10,11].

### Photodynamic Therapy

Photodynamic therapy is a method increasingly used in medicine. Its therapeutic effect is based on the administration of a photosensitizer into the body, which has the ability to absorb light [12,13,14]. Choosing the right light beam is a very important aspect, as different PSs show different absorption bands of given light lengths [15,16,17]. For example, protoporphyrin IX shows the highest absorption in the 405–415 nm range. The second peak of increased light absorption is in the 500–630 nm range, and the last is at 635 nm [18]. The use of PSs with long absorption bands should be pursued, as this ensures high tissue penetration and a high ability to induce excited states; the optimal absorption band is between 650 nm and 850 nm [15,19].

We can divide the compounds used in PDT according to their chemical structure. The first group are phenothiazine dyes, which include, among others, methylene blue; the second are phthalocyanine dyes, whose representative is zinc phthalocyanine. The third group—porphyrins—currently includes the most widely used compounds in PDT, namely 5-aminolevulinic acid (ALA) and its methyl ester, methyl aminolevulinate (MAL). The fifth, sixth and seventh groups are chlorites, xanthines and monoterpenes, respectively [20,21,22].

Through the excitation of photosensitizer particles with light, the destruction of pathological cells takes place. This process occurs by two different mechanisms. The first involves the transfer of electrons between the excited photosensitizer molecules and the surrounding cells, resulting in the formation of free radicals and anion radicals of the substrate and PS, which interact with oxygen molecules, leading to the generation of free oxygen radicals (ROS) [12,15,23]. In the second mechanism, there is a direct transfer of energy from the excited photosensitizer molecules to the oxygen molecule, which is in the basic energy state, resulting in the formation of so-called singlet oxygen characterized by strong oxidative properties [12,24,25]. Following these mechanisms, severe oxidative stress is produced, which leads to apoptosis and cell necrosis caused by damage to basic cellular structures [15,26]. PDT interferes with the vascularization systems of abnormal structures, leading to vascular closure, which generates hypoxia and subsequent cell death. PS molecules have a special ability to accumulate in newly formed vessels. Neovascularization is a process characteristic of cancerous lesions, and its arrest by PDT can be a major factor limiting tumor growth and metastatic capacity [27,28].

Thanks to the high affinity of photosensitizer particles for pathological cells, photodynamic therapy is a highly selective therapeutic method that spares healthy cells. In addition to its cytotoxic effect, PDT stimulates the immune system, inducing a local strong anti-inflammatory response [6,12]. Damage to cell membranes induces an increase in inflammatory cytokines and the complement system, which attack and remove dying cells through increased concentrations of macrophages and dendritic cells [29]. Photodynamic therapy is a non-invasive procedure, so it is possible to use it in an outpatient setting [30,31]. The effectiveness of PDT depends on the type of photosensitizer used, the dose, the site of action and the type of tumor [32].

Currently, it is mainly used for cancer. In oncology, combinations of PDT with other treatment methods—radiotherapy, surgical methods and even standard anticancer drugs—are used. It is used for cancers of the skin, head and neck, gastrointestinal tract, urinary tract and brain [33,34,35]. It can also be an effective therapeutic option in a range of non-cancerous conditions such as acne, solar keratosis and infections with drug-resistant bacteria [6,18].

## 2. Photodynamic Therapy in the Treatment of Atherosclerosis

**Figure 1:**
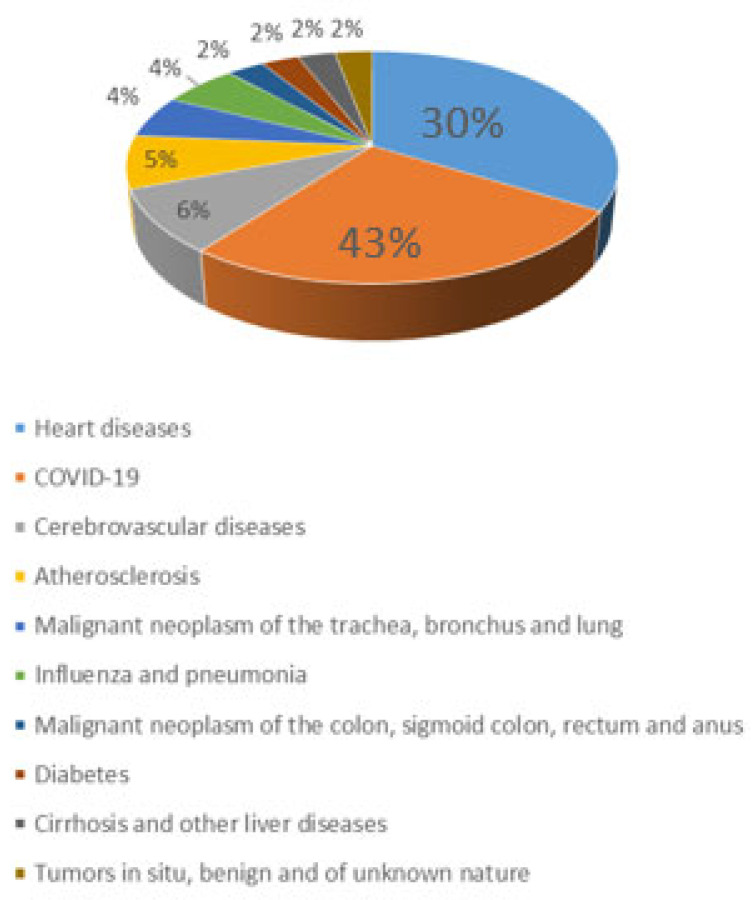
Causes of deaths in Poland’s population in 2021.

Previously, atherosclerosis was thought to be a problem that occurs primarily in the industrialized world, but as of today, it covers the entire world. Improved sanitation and the introduction of mandatory vaccinations have reduced the incidence of infectious diseases. However, the number of people suffering from chronic diseases, including atherosclerosis, has increased. At this point, atherosclerotic cardiovascular diseases are the most common cause of most deaths worldwide. Therefore, there is an urgent need for a thorough understanding of the genesis of this disease, the management of its treatment and control and the development of options to mitigate its consequences. It has been reported that in terms of cardiovascular disease, hypertension is the strongest risk factor that often contributes to death [36]. Based on data from the Central Statistical Office, approximately 130,000 people in Poland were predicted to die as a result of cardiovascular disease in 2021 (Figure 1).

Atherosclerosis is a chronic degenerative–inflammatory disease affecting the vessels, in the course of which endothelial cells are damaged, which then leads to an increased inflammatory process, the adhesion of blood morphotic elements, the proliferation of muscle cells and the accumulation of lipids. The disease mainly affects the aorta, coronary arteries, cerebral arteries and vessels in the extremities. The disease is asymptomatic for a long time [37,38,39]. 

**Figure 2:**
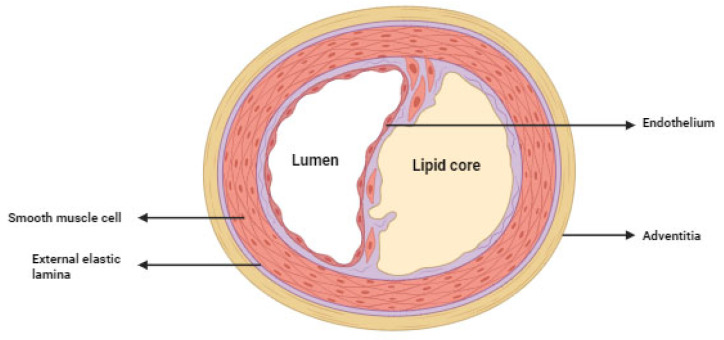
Atherosclerosis intermediate lesion. Restriction of blood flow in a vessel occurs when the resulting atherosclerotic plaque narrows it by at least 50% (this is known as hemodynamically significant stenosis). In contrast, a narrowing of the artery lumen by >80% (this is known as critical stenosis) can cause ischemia already at rest. If the atherosclerotic plaque enlarges significantly, or ruptures and consequently forms a thrombus on its surface, it usually leads to complete occlusion of the vessel, resulting in myocardial infarction.

Atherosclerosis is localized mainly in the inner membrane of many medium-sized and large arteries, especially at the sites of vascular division. This is most likely influenced by the nature of blood flow, as areas exposed to normal shear stress appear to be protected; here, endothelial cells express genes that protect against atherosclerosis [40]. The appendage may also play a role in the development of atherosclerosis and is characterized by lymphocyte infiltration (Figure 2) [41].

Atherosclerosis reduces blood flow through the stenosis, thus causing cardiovascular disease; however, the main mechanism is atherosclerotic thrombosis, which involves plaque damage due to the action of pro-inflammatory cytokines or chemokines on the fibrous cap. This causes plaque damage and rupture, and the prothrombotic material then becomes subject to the clotting system. Studies have indicated that LDL, a molecule that is surrounded by a characteristic component of apolipoprotein B, causes atherosclerosis [42,43]. Therefore, if the general population could maintain LDL concentrations that are comparable to those in newborns, atherosclerosis would become an orphan disease [44]. Despite the control of LDL cholesterol, blood pressure and other known risk factors, this is a high residual risk of atherosclerotic cardiovascular disease [45]. One of the most recent clinical trials of new cardiovascular medications, which were conducted on patients optimally treated with standard essential therapy, showed that about 1 in 20 patients is observed to have a recurrent ischemic episode within a year after an acute coronary syndrome [46,47]. In addition, one in ten patients who undergo an acute myocardial infarction in the U.S. will have to be re-hospitalized within one month, and this is associated with large costs of a personal and social nature [48].

Cohort studies, which include the Framingham study, have illustrated risk factors for atherosclerosis that were considered “traditional” [49]. However, the traditional factors do not currently reflect modern perceptions of atherosclerosis at this time. Genetic risk assessment is being improved on an ongoing basis and includes an increasing number of heritable variants with implications for atherosclerotic disease. Genetic panels have the ability to predict risk from birth and thus can help in the early targeted allocation of preventive measures in younger individuals with an increased genetic predisposition to atherosclerotic disease [50]. Lifestyle-related measures significantly reduce the risk of cardiovascular disease in terms of estimated genetic risk. However, the ability of new genetic risk scales to improve prediction of atherosclerotic disease is controversial [51,52].

**Figure 3:**
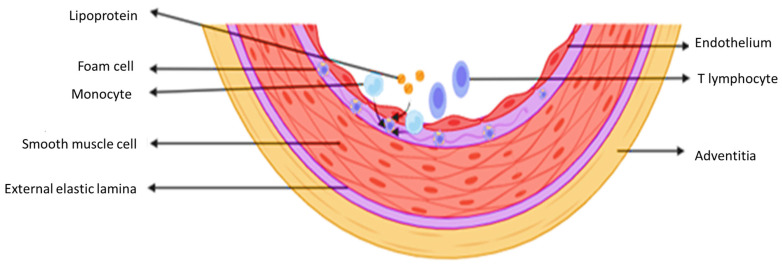
Initiation of atherosclerosis. Elevated LDL cholesterol is a major risk factor for the genesis of atherosclerosis. In addition, oxidized LDL plays a very important role in the genesis and in the entire progression of my-atherosclerosis in relation to native LDL.

Most reviews on atherosclerosis point to the key role of oxidized LDL as the main driver of the disease (Figure 3). Although LDL participates in atherogenesis, based on a large number of animal studies, there is little evidence to support a causal role for oxidized LDL in humans. Different types of clinical trials, referring to interventions with either antioxidant vitamins or one highly effective lipophilic anti-oxidant drug, have not shown a reduction in the incidence of complications of atherosclerosis. Recent studies support the involvement of cavelolin-1-dependent transcytosis of LDL by the endothelium in experimental atherosclerosis [53].

At this point, how LDL causes atherosclerosis is not completely understood. Oxidized lipids bind to plasminogen, and these lipids can activate fibrinolysis [54]. This results in the possibility that oxidized lipids may have a positive effect on atherogenesis, but also enhance thrombolysis, an opposite effect that contributes to the complete lack of benefit in antioxidant strategy studies [55]. Macrophages in plaques take up aggregated LDL during the process, while a protein that is linked to the LDL receptor can mediate the update of aggregated LDL through smooth muscle cells of the inner membrane [56]. Experimental and human observations provide confirmation that the recruitment of leukocytes in the blood involving the activation of endothelial cells, which line the arterial lumen, occurs early in lesion formation (Figure 3). In an atherogenic environment, endothelial cells often express leukocyte adhesion molecules, mediating the coiling and firm adhesion of white blood cells to the surface from the inside. Chemokines, on the other hand, direct the migration of leukocytes toward the arterial inner membrane. Mononuclear phagocytes proliferate in the area of the inner layer, which is the site of injury initiation [57]. These cells then absorb lipids, which causes them to become foam cells, a characteristic of atherosclerotic lesions. In the case of T lymphocytes, driving the acquired immune response, they interact with innate immune cells in the inner membrane area [58,59]. The pro-inflammatory group of monocytes influences the formation of macrophages with damage [60,61]. Bennett et al. realized a study that involved genetic lineage tracing, and they confirmed the origin of many foam cells in smooth muscle in mouse atherosclerosis [62].

As mentioned earlier, atherosclerosis is a major cause of cardiovascular and cerebrovascular disease [63]. Vascular smooth muscle cells (VSMCs) are located in the membrane of the vascular center [62]. VSMCs typically exhibit a contractile phenotype, characterized by a high degree of differentiation, but also have little or no ability to proliferate or migrate. Their main functions include maintaining vascular elasticity, vasoconstriction and regulating blood pressure [62]. When either the blood vessels are damaged or stimulated by oxidized LDL, the normal VSMC contraction phenotype is changed to an abnormal synthetic phenotype, characterized by a low degree of differentiation. This leads to VSMC migration toward the inner membrane and phagocytosis of cholesterol. The consequence is lipid accumulation and foam cell formation, which ultimately reduces plaque stability and accelerates the development of atherosclerosis [64,65,66,67].

Foam cells are a key starting point for the prevention and treatment of myoatherosclerosis. Studies have shown that only 30% of these cells in central and distal atherosclerotic plaques are derived from macrophages, while more than 40% are derived from VSMCs [68,69]. Therefore, inhibiting the transformation of VSMCs to a synthetic phenotype and to foam-like lesions plays an important protective role in the prevention and treatment of atherosclerosis.

The combination of basic research and clinical trials will significantly change traditional concepts of atherosclerosis and improve the ability to manage atherosclerosis risk.

One such method is photodynamic therapy. PDT can be used both as a diagnostic and therapeutic method [70]. PDT is a novel approach to treating diseases using photosensitizers and laser activation [71]. Irradiating the target tissue with a given wavelength can activate photosensitizers that are selectively accumulated in the target tissue and then induce photochemical reactions that promote autophagy, apoptosis and necrosis of diseased cells located in the tissue [71]. Atherosclerotic plaque is largely composed of low-density lipoproteins (LDL), to which photosensitizer molecules bind in a highly selective manner. In clinical studies conducted by Hayase et al. and Waksman et al., it was shown that PDT damages virtually the majority of atherosclerotic plaque cells while paradoxically causing stabilization of the plaque structure. This prevents its rapid disintegration and changes in normal vascular structure. The stabilization is likely due to PDT’s reduction of macrophages and foam cells in the atherosclerotic plaque. As evidenced by Peng et al. in a study conducted on rabbits, ALA reduces the number of macrophages by 64%, increases collagen content by 44% and causes a final increase in smooth muscle content by 18% within the atherosclerotic plaque [72,73,74,75].

Jain et al. showed that atherosclerotic plaques have a characteristic effect on the absorption and retention of curcumin [72]. Therefore, the synergy between curcumin and photodynamic therapy may become a novel idea for the prevention and treatment of atherosclerosis.

Curcumin (CUR), which is a polyphenolic compound extracted from the rhizome of Curcuma longa, has anti-cancer, anti-inflammatory, antioxidant, anti-atherosclerosis, anti-lipid and other pharmacological and photosensitizing activities [76,77,78,79].

Treatment of atherosclerosis is mainly pharmacological [80]. In patients who develop pain symptoms at rest, or when non-healing ulcerations or ceaseless chromias occur despite pharmacological or rehabilitative treatment, mechanical revascularization treatment using surgical and endovascular procedures should be implemented [81,82]. Endovascular surgical procedures are associated with an increased risk of complications in the form of restenosis due to endothelial and smooth muscle cell hyperplasia [83]. PDT also appears to be a good therapeutic option for the prevention and treatment of restenosis after the mechanical treatment of atherosclerotic plaques [10,84].

PDT has already been used to treat atherosclerosis in recent years, but studies have focused primarily on macrophages [85]. VSMCs are a very important component of the vessel wall, and they are actively involved in the development of atherosclerosis [62]. Wang et al. elucidated the regulation of PDT on autophagy and determined its specific role in VSMCs. In addition, they established an in vitro model of atherosclerosis to study [86].

Oxidized LDL has been linked to the development of atherosclerosis [87,88,89]. Wang et al., on the other hand, found that LDL induces the transformation of VSMCs in a synthetic phenotype which promotes their migration and foaming, confirming previous reports [89,90]. 

Studies have shown that PDT has anti-tumor effects and inhibits atherosclerotic plaque formation through the induction of apoptosis [91]. In addition, PDT can also induce autophagy in cells as a result of oxidative stress [91,92,93]. 

Other studies related to atherosclerosis have also confirmed that PDT can promote cholesterol excretion as a result of the induction of autophagy in THP-1 macrophages and by reducing macrophage transformation in the foam cell area [85].

### 2.1. Photodynamic Therapy as a Prevention of Coronary Restenosis after Surgical Correction

Balloon angioplasty is a highly effective method for treating vascular stenosis [94]. The procedure involves inserting a balloon via a catheter into the narrowed vessel and then inflating it with appropriate pressure to dilate the narrowed area. The method is used to unblock vessels in myocardial infarction, stable coronary artery disease and ischemic stroke, among others [95,96,97]. A complication that frequently occurs after balloon angioplasty is restenosis, which is the gradual re-growth of stenosis within a site that was previously treated with balloon angioplasty or a stent [98]. Restenosis results from endothelial cell hyperplasia and deposition of platelets, neutrophils and monocytes. Then, their place is taken by chronic inflammatory cells, namely macrophages and dendritic cells. Over time, intima cell hyperplasia begins to dominate as the main repair process [83,99]. During damage to the cells of the inner vascular membrane, chronic inflammation is produced, during which numerous changes also occur in the periphery of the muscles that build the vascular wall. Smooth muscle cells proliferate and migrate within the intima, leading to hyperplasia [100,101,102].

In the balloon angioplasty technique, access is from the venous side of the circumflex. This is because it has been documented that routine arterial puncture is associated with a much higher risk of complications, while longer monitoring is required after the procedure [103,104]. A standard wire and a catheter under constant fluoroscopy guidance is used. The size of the balloon is dated to the diameter of the referent vessel (1:1 ratio) [105]. For arteriovenous fistulas (AVF), balloons from 7 to 10 mm in diameter are used. Balloons that have a smaller diameter have a resource for treating arterial anastomotic stenosis in order to avoid excessive expansion of the surgical anastomosis. 

As for the length of the balloon, it is selected in relation to the length of the lesion being treated. Pressure is also an important consideration. Standard high-pressure balloons can withstand pressures of 20 atm, but newer balloons are capable of producing pressures as high as 40 atm. In terms of treatment AVF, higher pressures are much more often required compared to arteriovenous grafts (AVG). The average pressure that is required for successful balloon waist closure is from 15 to 17 atm [106,107].

There are studies that have attempted to examine the effect of inflation times on outcomes and long-term patency, but they have not been clear [108,109].

A technically successful angioplasty is defined as achieving less than 30% residual stenosis. In situations where multiple stenosis occur, treatment of all lesions contributing to clinical dysfunction is undertaken [110]. Dysfunction is a blood flow rate of less than 600 mL/min [111].

It is known that balloon angioplasty and stents are, at this moment, widely used in the clinical treatment of patients with coronary artery disease. However, the vessel lumen often undergoes further stenosis. This is observed up to 6 months after treatment of mechanical damage caused by stent implantation or balloon angioplasty. Therefore, the incidence of restenosis is about 10% [112,113,114]. The mechanism of restenosis is similar to that of wound healing [115,116].

It has been shown that PDT can prevent restenosis by inducing apoptosis of smooth muscle cells. Photosensitizers have varying degrees of binding to smooth muscle cells. Indocyanine green (ICG), zinc phthalocyanine, protoporphyrin IX and chlorin e6 are the most captured by the muscles. The ways in which PDT affects muscle cells are not fully understood; their effect on caspase activation via mitochondrial pathways is suggested. Another pathway for inducing apoptosis is through effects on the endoplasmic reticulum [10,26,117].

Photodynamic therapy also leads to the deactivation of basic fibroblast growth factor (FGF-β). Inactivation of FGF-β leads to the inhibition of muscle cell proliferation. In a study conducted on rabbits by Waka-matsu et al., it was shown that sodium thalaporphin, even at a low dose of 10 J/cm^2^, can cause the complete inhibition of proliferative capacity as early as two days after application of the photosensitizer [72,118]. In a clinical trial conducted by Jenkins et al. on seven patients undergoing angioplasty, the positive effect of photodynamic therapy in preventing restenosis and its safety were confirmed; the only reported side effects were nausea and facial erythema [119,120,121].

Ortu et al., using phthalocyanine; Eton et al., using photofrin; Asahara et al., using hematoporphyrin; and Hsiang et al., using hematoporphyrin, confirmed in their animal model studies that PTD administered early (up to 1 week after damage, up to 1–2 weeks according to Asahara et al.) in case of damage to the inner layer of the vascular membrane can effectively inhibit endothelial cell hyperplasia, reducing the process of restenosis [119]. In a study conducted on 15 rats, the possibility of using PDT to inhibit endothelial membrane hyperplasia in carotid arteries during balloon angioplasty was tested. The control group consisted of rats that underwent sham surgery. The study group was divided into rats undergoing balloon angioplasty only, those undergoing balloon angioplasty and one dose of PDT irradiation—7 days after the procedure, and balloon angioplasty and two doses of PDT irradiation—7 and 14 days after the procedure. The study used ICG, which was administered one hour before light irradiation. Parameters, such as thickness of the vascular wall, area of the arterial wall and diameter of the arterial lumen, were then studied. In the group in which a double dose of PDT was used, complete inhibition of restenosis after angioplasty was detected. The studied parameters of the carotid arteries were similar to those observed in the control group. Similar observations occurred in the group where only a single dose of PDT was used [120,122,123,124].

### 2.2. Application of Photodynamic Therapy in the Treatment of Atrial Fibrillation

Atrial fibrillation (AF) is the most common heart rhythm disorder involving rapid and uncoordinated stimulation of the atria. It is predicted that, in 2050, there will be about 5.6 million AF patients in the U.S. [125,126], and by 2030, there will be 14–17 million patients with the condition in the European Union, with 120,000–215,000 new patients newly diagnosed annually [127,128,129]. According to estimates, the prevalence of AF in adult patients (20 years or older) is about 3% [130,131]. A higher incidence is found in much older individuals [132] and in patients who additionally have conditions such as hypertension, heart failure, coronary artery disease (CAD), valvular heart disease, have obesity, diabetes or chronic kidney disease (CKD) [129,133,134,135,136].

A significant increase in the prevalence of AF is associated with the better detection of silent AF [137,138,139], with increasing age and with conditions predisposing to AF [140].

In addition, AF is independently associated with a two-fold increased risk of death from any cause in women and a 1.5-fold increased risk in men (Table 1) [141,142,143,144,145].

The pathophysiology of atrial fibrillation involves microreentry waves, within which stimulation hits tissue permanently capable of stimulation, which leads to permanent electrical activity of the atria. The pathophysiological significance is also given to the electrical impulses generated in ectopic stimulating centers. In AF, the ectopic center is most commonly located within the pulmonary veins. Impulses can also be generated within the inferior vena cava, coronary sinus, border crest and free wall of the left atrium [146,147,148]. In people with atrial fibrillation resistant to pharmacological treatment, percutaneous ablation is used, which is a very effective therapeutic method used in symptomatic patients [149].

In an animal model study, the effectiveness of ablation of the ectopic medium within the superior vena cava with PDT was proven. As PS, talaporfin sodium was used, and light with a wavelength of 663 nm was used to excite it. In all seven tested dogs, electrical insulation was successfully performed without any complications [150,151].

Ablation with PDT holds particular hope for the treatment of ectopic centers located within sites characterized by high vascular flow. At these sites, the use of standard ablation methods is often associated with failure. PDT seems to be a good therapeutic option, as well as relatively easy to perform in contrast to traditional methods [151].

## 3. Current Restrictions Related to the Use of PDT

One of the significant limitations of the use of PDT is the size of the area in which it is applied. In the case of a larger area of lesion, in order to maintain the same depth of penetration and thus the effectiveness of treatment, the irradiation power should be increased, which significantly limits the possibility of applying PDT over large areas [152,153]. In addition, there may be a potential expansion of the irradiated area and an improvement in the conditions of the tumor microenvironment [154]. By inducing changes within cells, PDT leads to necrosis and apoptosis. Necrosis is an immediate process, while in the case of apoptosis, there is a certain delay which results from the activation of enzymatic processes and the cascade of proteins within the cell [155]. Cancer cells can predict survival mechanisms that will allow them to develop resistance to PDT [154]. PDT requires research in the field of optimizing the conditions in which the therapeutic process is to be carried out in order to maximize the limitation of the change and, consequently, the therapeutic effect [156].

Problems in the use of PDT in the treatment of lesions within the coronary vessels arise from the difficulty in reaching the beam of light directly into the vessel, as they are obscured by the ribs and surrounding skeletal muscles. Treatment of atherosclerotic plaques also requires further research in the optimal range of photosensitizer concentrations in the body, the type of compound used and determining the best wavelength and light source. Unfortunately, it turned out that many of the photosensitizers used are toxic and can cause severe allergic reactions [152,153,157]. In Table 2, a comparison of the features of current ablation and PDT is presented.

### Future Perspectives

As is well known, atherosclerosis arises at the nanoscale. Therefore, nanotechnology may be a promising prospect for molecular imaging as well as treatment methods for atherosclerosis [158]. Nanoparticles have the ability to increase stability, solubility in water and to absorb diagnostic agents or various types of therapeutic compounds and additionally extend their circulation time [159]. Although nanomedicine is of great interest in cancer therapy, its use in the treatment or prevention of atherosclerosis is understudied. A study that emerged as one of the first in the 2000s showed that nanoparticles that targeted fibrin detected clots, and it is randomly likely that they were sensitive to lesions [160,161]. Another study talks about the use of superparamagnetic iron oxide nanoparticles for imaging atherosclerotic lesions (animal model) [162].

Some of the most widely studied nanoparticles for the treatment of atherosclerosis are polymeric nanoparticles (range from tens to hundreds of nm). In particular, attention is focused on poly(d,l-lactic-co-glycolic acid) nanoparticles due to the fact that they have excellent biocompatibility and biodegradability [163,164,165,166,167,168]. The surface of such nanoparticles can be derivatized using a wide range of targeting ligands (e.g., S2P peptide 52, RGD peptide 141) or biomimetic materials (e.g., erythrocyte membrane, exosomes, extracellular vesicles) to target or carry out preferential accumulation in plaque macrophages or another type of polymer to prolong their circulation in the blood [169,170,171,172,173,174].

HDL-like nanoparticles also seem very promising. HDL (7–13 nm) is composed of phospholipids and apolipoprotein AI (apoA-I). It can carry cholesterol from lipid-laden plaque macrophages to the liver during the reverse cholesterol transport process. It takes a long time to extract apoA-I from human plasma; various genetic variants of apoA-I or recombinant apoA-I have been developed to be able to replace apoA-I from human plasma to obtain reconstituted HDL nanoparticles or HDL designed to protect against atherosclerosis similar to HDL itself. HDL nanoparticles are being explored for nanocarriers to be used either for delivery of therapeutic agents or for imaging to treat atherosclerosis [175,176,177,178,179,180,181,182,183].

Studies on the renin-angiotensin-aldosterone system (RAAS) have shown that it has a very important role in the area of regulation of the inflammatory response. It is tasked with managing the recruitment of inflammatory elements toward the site of injury. Inflammatory cells have the ability to produce angiotensin II, which contributes to local RAAS stimulation, sustaining the inflammatory cycle [184]. Angiotensin II is a local and biologically active mediator that affects VSMCs and endothelial cells. It has a role of an endocrine nature with effects on the kidneys and on hemodynamics [185]. Angiotensin II is also a known regulator of various molecules that are very important for the development of inflammation, which are cytokines, chemokines, growth factors and adhesion molecules. Accordingly, angiotensin II could promote atherosclerotic plaque development in terms of increasing the expression of VCAM-1, ICAM-1 and P-selectin [186].

Angiotensin-1-7 stimulates anti-inflammatory phenotypes, resulting in the inhibition of lipid accumulation in the vascular area [187]. Studies on ApoE-KO mice have shown that using Ang-1-7 treatment, macrophage infiltration is reduced, and oxidative stress occurs as a result of the reduction of Nox4, which is an important subunit of the entire NADPH oxidase complex [188]. Based on other studies, it comes out that the expression of TNF-α, IL-6 and other pro-inflammatory cytokines significantly decreased in response to Ang-1-7 administration, and that in aortic plaque and macrophages significantly decreased with ApoE-KO [189]. Pretreatment through the MasR agonist AVE0991 resulted in decreased levels of IL-2 and activated CD4+ T cells [190,191]. The above data establish the protective actions of the Ang-1-7/MasR pathway against atherosclerosis. Treatment through Ang-1-7 showed, firstly, a reduction in layer growth in terms of new inner membrane due to structural restoration of the endothelium and, secondly, the appearance of atherosclerosis-protective properties was observed due to AT2R and MasR binding [192]. Ang-1-7 also reduces atherosclerotic lesion formation due to AT2R activation, which provides low collagen accumulation. According to the study, however, Ang-1-7 administration leads to collagen accumulation, which consequently increases plaque stability [188]. Thus, treatment through A77, which is an Ang-1-7 antagonist, promotes low plaque stability and low collagen levels [187].

The use of photodynamic therapy in the treatment of various pathological conditions brings very promising prospects for the future. Unfortunately, the number of studies is currently very small and cannot provide reliable data. The amount of research should be increased, both on animal models and clinical trials on humans, in order to intensify the whole process, determine the best types of photosensitizers for a given lesion and, consequently, the best light lengths [193]. It is necessary to standardize and establish strict treatment regimens and medical procedures involving the combination of photodynamic therapy methods with other therapeutic methods in order to increase its effectiveness, as well as to determine specific photosensitizers and their doses and light lengths that will ensure maximum effectiveness in a given disease entity [157,194,195].

One of the major limitations currently faced by PDT is the delivery of PS and a beam of light radiation within the lesion. Currently ongoing research on the system of biomaterials transferring PS molecules into the immediate vicinity of the lesion brings some hope [196]. Also here, further research is needed on the biodegradability of nanoparticles, their chemical structure, specificity for given types of photosensitizers, uptake of nanoparticle-sensitizer complexes and their cytoxicity [197]. Better methods of delivering PS particles to the disease focus will provide better penetration and perhaps reduce the required dose of irradiation while maintaining the same therapeutic effect [198].

In recent years, porphyrins, chlorines and dye-based photosensitizers have been studied and may be used in the treatment of atherosclerotic plaques (Table 3) [199,200,201,202,203,204,205,206,207,208,209,210,211,212,213,214,215,216,217,218,219,220,221,222]. The results showed that the vast majority of porphyrin-based photosensitizers accumulate in atherosclerotic plaques. This happens because the injected porphyrins quickly bind to blood proteins, mainly low-density lipoproteins. One study showed the increased selectivity of the uptake of a benzoporphyrin derivative located in the atherosclerotic plaque of rabbits thanks to the initial combination of the benzoporphyrin derivative with a low-density lipoprotein [223,224].

It is necessary to further search for compounds that could potentially be used as photosensitizers and to determine all side effects, pharmacokinetic and pharmacodynamic parameters, interactions with other compounds and the bioavailability of already used photosensitizers. It is also necessary to determine the best environmental conditions in which photodynamic therapy should be used [193,197].

## 4. Conclusions

PDT is a therapeutic method that has become increasingly popular in recent years. It is increasingly used in various fields of medicine. Currently, it is most often used in cancers located mainly in the central nervous system (CNS). More and more reports refer to the use of PDT in cardiology. Current research is ongoing on the treatment of atherosclerotic plaques, the prevention of restenosis after mechanical treatment and the ablation methods of ectopic centers in the treatment of atrial fibrillation.

It should be noted that there have been significant improvements since PDT was first used to treat atherosclerosis. The vast majority of problems or drawbacks of an environ-mental or technological nature have either been solved or are constantly being worked on. There are new generations of photosensitizers, characterized by higher specificity and which are more accurate in terms of their distribution in the atherosclerotic plaque. In addition, all technologies of local and intravascular drug delivery along with light have resulted in a reduction of adverse effects by which the interest in PDT has greatly increased. Through the method of local delivery, contact time is significantly reduced, and a reduction in adverse effects is observed. This makes PDT suitable for clinical use. Therefore, the development of new types of “perfusion balloon catheters” will create the possibility of effective drug delivery in relatively short periods of time and using very high drug concentrations. Somehow, the new catheters eliminate most of the problems resulting from insufficient drug selectivity or lumen, but unfortunately, some issues are still standing for the time being, such as the optimization of PDT components, response to PDT, insufficient clinical data, etc.

PDT can therefore be widely used in two cases. The first is the treatment of secondary vulnerable plaques. It is observed that after stent implantation, about 14% of patients develop plaque rupture. In addition, the progression of untreated coronary plaques becomes quite a burden. Although detection of vulnerable plaque has improved, there is a need for preventive therapy. PDT has the ability to stabilize plaque; hence, the said sensitive plaques may be a target for PDT. In addition, systemic administration of the photosensitizer offers the possibility of treating multiple vascular segments within the scope of a single intervention, which is another plus of this solution. A second use case for PDT may be the prevention of “neo-atherosclerosis,” which follows the use of coronary stents. Thus, PDT’s ability to stabilize atherosclerotic plaque can be used to prevent rupture of neo-atherosclerotic lesions. The use of PDT immediately after percutaneous coronary intervention will offer the possibility of preventing the recurrence of restenosis that was induced by the stent.

Both clinical and preclinical studies are promising, and there is a need to initiate further studies of the use of PDT in the treatment and prevention of myo-atherosclerosis. Analysis of PDT parameters and their improvement will bring PDT into the realm of routine clinical practice.

## Figures and Tables

**Table 1:** Morbidity and mortality associated with atrial fibrillation.

Cause	Association with AF	References
Death	Increased mortality, heart failure or stroke.	[141,142,143,144]
Stroke	About 20–30% of all strokes are a consequence of atrial fibrillation. It is observed that an increasing number of stroke patients are being diagnosed with paroxysmal atrial fibrillation.	[141]
Hospitalizations	About 10–40% of AF patients are hospitalized within one year.	[144]
Quality of life	This is reduced in patients with atrial fibrillation regardless of whether they have other cardiovascular conditions.	[141,142,143,144,145]
Left ventricular dysfunction/ heart failure	Left ventricular dysfunction is diagnosed in about 20–30% of all patients who have been diagnosed with AF. This has the effect of exacerbating left ventricular dysfunction in many AF patients.	[143]
Cognitive decline and vascular dementia	Deterioration of functions of a cognitive nature can also appear in patients with AF who are treated with anticoagulants. It is observed that changes in the white matter of the brain occur more frequently in patients with AF compared to patients without AF.	[141]

AF—atrial fibrillation.

**Table 2:** Advantages and disadvantages of PDT ablation compared to radiofrequency ablation (RF ablation).

Factor	PDT	RF	Defect of PDT	References
Heat	Independent	Dependent	-	[151,152]
Cooling	Unnecessary	Required	-	[151]
Edema	No	Yes	-	[151]
Thrombus formation	No	Yes	-	[151]
Steam pop	No	Yes	-	[151]
Local blood flow	Independent	Affected	-	[151]
High impedance sites	Applicable	Inapplicable	-	[151]
Lesion size	Governed by light penetration	Governed by temperature	Light penetration limitations	[151,155]
Mechanism of injury	Necrosis and apoptosis	Heat injury	Potential late expansion of the lesion	[151,154]
Ablation time	Longer	Shorter	Longer ablation time	[151]
Photosensitizer	Yes	No	Risk of systemic adverse effects	[151,152]

PDT—photodynamic therapy; RF—radiofrequency.

**Table 3:** Treatment and prevention of atherosclerosis based on the use of PDT.

Photosensitizer	Absorption Spectrum (nm)	Doses Used and Animal Models	Plate Localization Time/Drug Light Interval	Treatment of AS	Conclusions	References
5-aminolevulinic acid, Levulan	630	60–120 mg/kg, intravenous administration; animals: rabbits, pigs	1.5–48 h, plaque: normal arterial ratio 9 to 12:1	External/intravascular PDT (30–150 J/cm^2^), 28-day follow-up	A reduction in the progression of atherosclerosis and macrophage content was observed. Moreover, it prevents intimal hyperplasia caused by the stent.	[72]
Hemato-porphyrin derivative, Photofrin, sodium porfimer	630	2.5 mg/kg, intravenous/topical administration; animals: rabbits, mini pigs. Studies also in a human artery with a stent	1–48 h, plaque: normal arterial ratio 3:1	External/intravascular PDT (27–120 J/cm^2^), follow-up 18 months/every 4 weeks–6 months	The accumulation took place in dental plaque. Studies have shown that PDT prevented intimal hyperplasia for up to 16 weeks and additionally did not cause side effects or initiate in-stent restenosis in humans.	[72]
Phthalocyanine derivative, Photosens	670	2.5–5 mg/kg; use in the rat carotid artery	20–30 min to 24 h	External PDT (50–100 J/cm^2^), follow-up for 6 months	The results show that it prevents neointimal hyperplasia for up to 6 months.	[72]
Talaporfin sodium, mono-L-aspartyl chloride e6 (NPe6)	664	2–5 mg/kg, intravenously; animals: rabbits	30 min–6 h	External/intravascular PDT (30–50 J/cm^2^), 14-day/25-week follow-up	Prevention of neointimal hyperplasia and premature destruction of the elastic fiber was observed.	[72]
Motexafin lutetium (MLu)	710–760	1.2–1.2 mg/kg, administration: intravenous/local perfusion; animals: rabbits	15 min–5 h, plaque: normal arterial ratio from 16 to 34:1	Endovascular PDT (180 J/cm^2^), 2-week follow-up	Studies have shown a reduction in macrophage burden and atherosclerosis.	[72]
Verteporfin, benzoporphyrin derivative monoacid ring A (BPD-MA)	692	2 mg/kg, administration: intravenous/local perfusion 25 mg/mL; animals: minipig, rats	15 min–1 h, plaque: normal arterial ratio 1.7 to 3.5	External PDT (100 J/cm^2^), 21-day/25-week follow-up	The results showed that it has a preventive effect on neointimal hyperplasia.	[72,75]

AS—atherosclerosis; NPe6—Mono-L-aspartyl chlorin e6; MLu—Motexafin lutetium; BPD-MA—benzoporphyrin derivative monoacid ring A.

## Data Availability

All data is included in manuscript.
